# Combined Genetic and Genealogic Studies Uncover a Large BAP1 Cancer Syndrome Kindred Tracing Back Nine Generations to a Common Ancestor from the 1700s

**DOI:** 10.1371/journal.pgen.1005633

**Published:** 2015-12-18

**Authors:** Michele Carbone, Erin G. Flores, Mitsuru Emi, Todd A. Johnson, Tatsuhiko Tsunoda, Dusty Behner, Harriet Hoffman, Mary Hesdorffer, Masaki Nasu, Andrea Napolitano, Amy Powers, Michael Minaai, Francine Baumann, Peter Bryant-Greenwood, Olivia Lauk, Michaela B. Kirschner, Walter Weder, Isabelle Opitz, Harvey I. Pass, Giovanni Gaudino, Sandra Pastorino, Haining Yang

**Affiliations:** 1 Thoracic Oncology Program, University of Hawai‘i Cancer Center, Honolulu, Hawai’i, United States of America; 2 Laboratory for Medical Science Mathematics, RIKEN Center for Integrative Medical Sciences, Yokohama City, Kanagawa, Japan; 3 Genealogy from the Hart, Honolulu, Hawai’i, United States of America; 4 Mesothelioma Applied Research Foundation, Alexandria, Virginia, United States of America; 5 Klinik für Thoraxchirurgie Universitätsspital, Zürich, Switzerland; 6 Department of Cardiothoracic Surgery, New York University Langone Medical Center, New York, New York, United States of America; Cleveland Clinic Genomic Medicine Institute, UNITED STATES

## Abstract

We recently discovered an inherited cancer syndrome caused by BRCA1-Associated Protein 1 (*BAP1*) germline mutations, with high incidence of mesothelioma, uveal melanoma and other cancers and very high penetrance by age 55. To identify families with the BAP1 cancer syndrome, we screened patients with family histories of multiple mesotheliomas and melanomas and/or multiple cancers. We identified four families that shared an identical *BAP1* mutation: they lived across the US and did not appear to be related. By combining family histories, molecular genetics, and genealogical approaches, we uncovered a BAP1 cancer syndrome kindred of ~80,000 descendants with a core of 106 individuals, whose members descend from a couple born in Germany in the early 1700s who immigrated to North America. Their descendants spread throughout the country with mutation carriers affected by multiple malignancies. Our data show that, once a proband is identified, extended analyses of these kindreds, using genomic and genealogical studies to identify the most recent common ancestor, allow investigators to uncover additional branches of the family that may carry *BAP1* mutations. Using this knowledge, we have identified new branches of this family carrying BAP1 mutations. We have also implemented early-detection strategies that help identify cancers at early-stage, when they can be cured (melanomas) or are more susceptible to therapy (MM and other malignancies).

## Author Summary

Germline *BAP1* mutations cause a cancer syndrome characterized by high incidence of mesothelioma (MM), uveal melanoma and other cancers, and by very high penetrance, as all individuals carrying *BAP1* mutations developed at least one, and usually several, malignancies throughout their lives. Through screening MM patients with histories of multiple cancers, we found four supposedly unrelated patients that shared an identical germline *BAP1* mutation. We investigated whether this *BAP1* mutation occurred in a ‘hot-spot’ for “*de novo*” mutations or whether these four MM patients shared a common ancestor. Using molecular genomics analyses we found that they are related. By genealogic studies we traced their ancestor to a couple that emigrated from Germany to North America in the early 1700’s; we traced the subsequent migration of their descendants, who are now living in at least three different US States. Our findings demonstrate that *BAP1* mutations are transmitted among subsequent generations over the course of centuries. This knowledge and methodology is being used to identify additional branches of the family carrying *BAP1* mutations. Our study shows that the application of modern genomic analyses, coupled with “classical” family histories collected by the treating physician, and with genealogical searches, offer a powerful strategy to identify high-risk germline *BAP1* mutation carriers that will benefit from genetic counseling and early detection cancer screening.

## Introduction

Malignant mesothelioma (MM) is frequent (up to 5% prevalence) in individuals who are heavily exposed to asbestos and/or other mineral fibers [1]. Moreover, we discovered that the risk of developing MM is transmitted in an autosomal dominant fashion in certain Turkish families, in which over 50% of family members developed MM [2]. In subsequent studies in US families with high incidence of MM and of uveal melanoma (UM) and no apparent exposure to mineral fibers, we identified germline mutations in the *BAP1* gene, as the major risk factor for MM and UM development [3]. Thereafter, we and others confirmed that germline *BAP1* mutations are a common heritable factor that predispose to MM, UM, cutaneous melanoma (CM), cholangiocarcinoma, renal cell carcinoma (RCC), and basal cell carcinoma (BCC) [4–6], and to benign atypical melanocytic lesions known as MBAITs [7, 8], and likely to several other malignancies including brain, breast, lung cancer, and sarcomas [9],—recently grouped together into the “BAP1 cancer syndrome” [7]. Thus, similarly to germline *TP53* mutations that cause the Li-Fraumeni syndrome [10], germline *BAP1* mutations are associated with a variety of cancers. There is, however, a preponderance of MMs and melanomas [7].

BAP1 is a deubiquitylase that associates in the nucleus with multi-protein complexes that regulate key cellular pathways, including transcription, DNA replication and the DNA damage response [9, 11, 12]. BAP1 tumor suppressor functions have been attributed to its ability to regulate gene transcription via (i) interaction to host cell factor-1 (HCF1), Ying Yang 1 (YY1), and E2F1 [13, 14], (ii) modulation of histone H2A ubiquitylation [15], (iii) maintaining DNA integrity [11, 16] and modulating DNA repair by homologous recombination [12, 16]. All germline *BAP1* mutations, identified so far, lead to inactive forms of BAP1, lacking deubiquitylating activity or to truncated variants that lack the nuclear localization signal. Therefore, it appears that, to function as a tumor suppressor, BAP1 must maintain both nuclear localization and deubiquitylating activity [17].

All carriers of germline *BAP1* mutations studied so far have developed at least one malignancy by age 55 and many developed multiple cancers [18]. Familial MMs in these individuals occur at a median age of 56.3 in either pleura or peritoneum (frequency ratio: 1/1), have a M:F ratio of 0.73:1 and are associated with prolonged survivals of 5–10 or more years; compared to a median age at diagnosis of 72, a 86%:14% pleural/peritoneal ratio; a M:F ratio of 4:1 and a median survival of <1 year in sporadic MM [18]. Thus, MM patients carrying germline *BAP1* mutations benefit from this information, and their relatives may benefit from screening programs for early cancer detection, when these malignancies can be cured by resection (melanomas) or are more susceptible to therapy (MM and other cancers).

## Results

### Detection of *BAP1* mutations in four apparently unrelated BAP1 families

**Fig 1:**
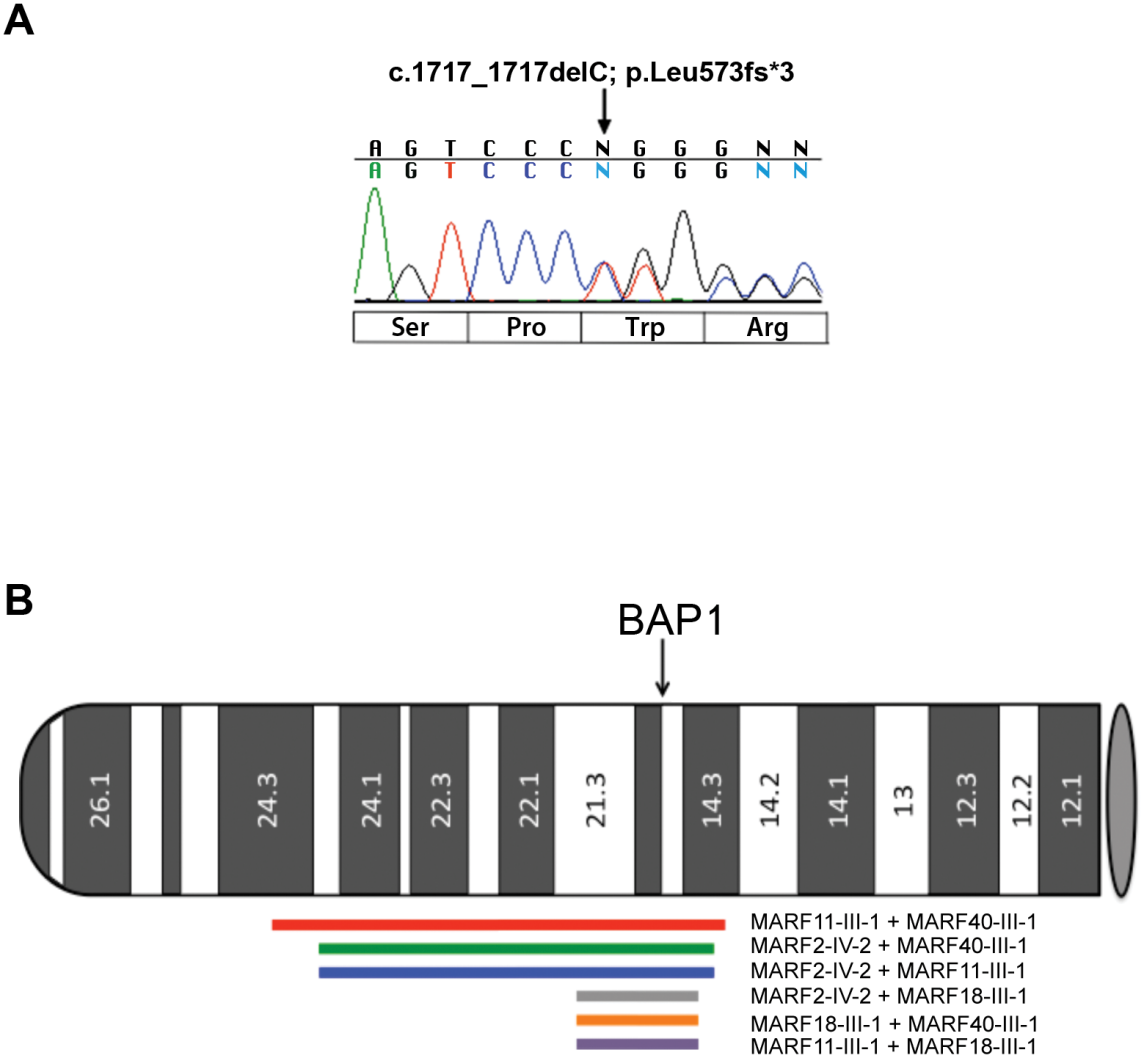
Electropherogram of BAP1 c.1717_1717delC mutation and chromosome 3 IBD shared haplotypes in the 4 founder MARF patients studied. **(A)** Representative electropherogram of germline *BAP1* MARF2-IV-2 founder mutation. The heterozygous C deletion at nucleotide position c.1717 of the *BAP1* gene is predicted to be a frame-shift mutation leading to a truncated protein of 573 amino acids in length. Nucleotide sequence is shown above the electropherogram and predicted amino acid changes are below. **(B)** Idiogram showing Identity By Descent (IBD) shared haplotypes of the DNA regions surrounding *BAP1* in the germline DNA of the 4 founder MARF patients studied. The p-arm of chromosome 3 depicts the position and extent of shared IBD haplotype segments (LOD>3) that overlap the *BAP1* gene. Red line: MARF11-III-1 + MARF40-III-1 (chr3:21.8–55.9, 34.2 Mbp, LOD = 176.6); green line: MARF2-IV-2 + MARF40-III-1 (chr3:25.55–55.12, 29.6 Mbp, LOD = 145.2); blue line: MARF2-IV-2 + MARF11-III-1 (chr3:25.55–55.12, 29.6 Mbp, LOD = 142.1); gray line: MARF2-IV-2 + MARF18-III-1 (chr3:45.40–54.5, 9.1 Mbp, LOD = 38.8); orange line: MARF18-III-1 + MARF40-III-1 (chr3:45.40–54.5, 9.1 Mbp, LOD = 38.1); purple line: MARF11-III-1 + MARF18-III-1 (chr3:45.40–54.5, 9.1 Mbp, LOD = 37.1). Gray oval represents the centromere.

Twenty-two MM patients were recruited based on family histories suggestive of the BAP1 cancer syndrome and selected according to the inclusion criteria described in the Methods section. None of the individuals, who met the inclusion criteria, reported a history of asbestos exposure. Sequence analysis of DNA isolated from peripheral blood mononuclear cells of these patients revealed that 4/22 of these familial MM cases, carried germline *BAP1* mutations. One patient with peritoneal MM carried a heterozygous *BAP1* variant (c.1938T>A, p.Tyr646*) in exon 15, leading to a stop codon and a truncated BAP1 protein, predicted to be 646 amino acids long and lacking the nuclear localization signal. The other three MM patients with germline *BAP1* mutations (MARF11-III-1, MARF18-III-1, MARF40-III-1) carried an identical mutation (*c*.*1717_1717delC*, p.Leu573fs*3, Fig 1A) in exon 13. MARF11-III-1 proband and family are from Maryland, MARF18-III-1 proband and family are from California, MARF40-III-1 proband and family are from Texas, and they were apparently unrelated. We previously found the same *BAP1* germline deletion in another apparently unrelated patient from Texas, MARF2-IV-2 (referred to as SP-002 in our previous study) [3]. Based on these results, we concluded that either *c*.*1717_1717delC* was a hotspot for “*de novo*” *BAP1* mutations or these four families had a common ancestor and *BAP1* mutation was transmitted across multiple generations.

Sanger sequencing revealed that the four probands sharing the *c*.*1717_1717delC BAP1* mutation also shared a rare allele of a synonymous SNP (rs71651686, minor allele frequency = 0.0016, according to NCBI dbSNP database) in exon 11, which is located 1770 bp upstream of the *c*.*1717_1717delC* variant in exon 13. Other than in the four probands, this allele was not found in any additional MM patient tested so far in our laboratory, including MM patients that were tested outside the current study. Eight individuals from the 1000 Genomes Project (1000G) have this rare SNP; however, they do not have the *BAP1 c*.*1717_1717delC* mutation. Moreover, the *BAP1 c*.*1717_1717delC* mutation is not present in any of the three genome-wide/exome-sequencing variant databases (1000G+UK10K+ESP: which include a total of 8286 genomes surveyed). The rare allele of synonymous SNP rs71651686 is unlikely to have any functional impact, but since the probability for any given individual to carry both the rare allele of rs71651686 SNP and the *c*.*1717_1717delC* variant was estimated to be less than 8.0x10^-7^, their presence together in the four individuals provided an initial indication that they were shared by descent from a common ancestor.

### Haplotype structure and linkage disequilibrium (LD) blocks

We investigated whether the mutation occurred “*de novo”* in separate unrelated family trees or whether it was inherited identical-by-descent (IBD) from a common ancestor. We genotyped these four MM patients sharing the *c*.*1717_1717delC BAP1* mutation and four unrelated healthy controls for 657,893 SNPs using the Illumina OmniExpress (OE) platform. We performed a population genetic and shared haplotype analysis of the data and we combined the SNP analysis with publicly available genotypes for 2141 samples from 19 worldwide population groups genotyped by 1000G [19] on the Illumina Omni 2.5M platform (Omni2.5).

We analyzed the four probands and four controls, together with the 1000G data analysis, using principal component analysis (PCA) to estimate the ancestral populations of our samples [20]. From the PCA analysis, we found that the four probands clustered closest to 1000G populations with ancestry from Central Europe or Great Britain (CEU, GBR; n = 205; S1 Fig).

Next, we analyzed our samples for ‘cryptic relatedness’, which is an unexpected relatedness between samples not known to be related based on family history [21]. We estimated relatedness between our samples and those from 1000G using a genome-wide IBD analysis [22, 23]. The results of the IBD analysis identified measurable relatedness only between the four MM patients. The most closely related samples were MARF11-III-1 and MARF40-III-1, which had a kinship coefficient of 0.0186, suggesting relatedness approximately equal to that of second degree cousins (S1 Table).

We then examined the haplotype structure in these 8 samples around the *BAP1* gene. We estimated phased haplotypes (i.e, clusters of tightly linked alleles along a chromosome) from our samples and those from the 1000G genotype data using the SHAPEIT2 [24] program. Analysis using BEAGLE [25] revealed that the only samples that shared significant haplotype segments (LOD>3; LOD = base 10 log of the likelihood ratio) spanning the *BAP1* gene were the four probands. Fig 1B depicts the pairwise extent of those shared segments, which ranged in length from 9.1 to 34.2 megabase pairs (Mbp).

### Tumor tissues analyses

**Fig 2:**
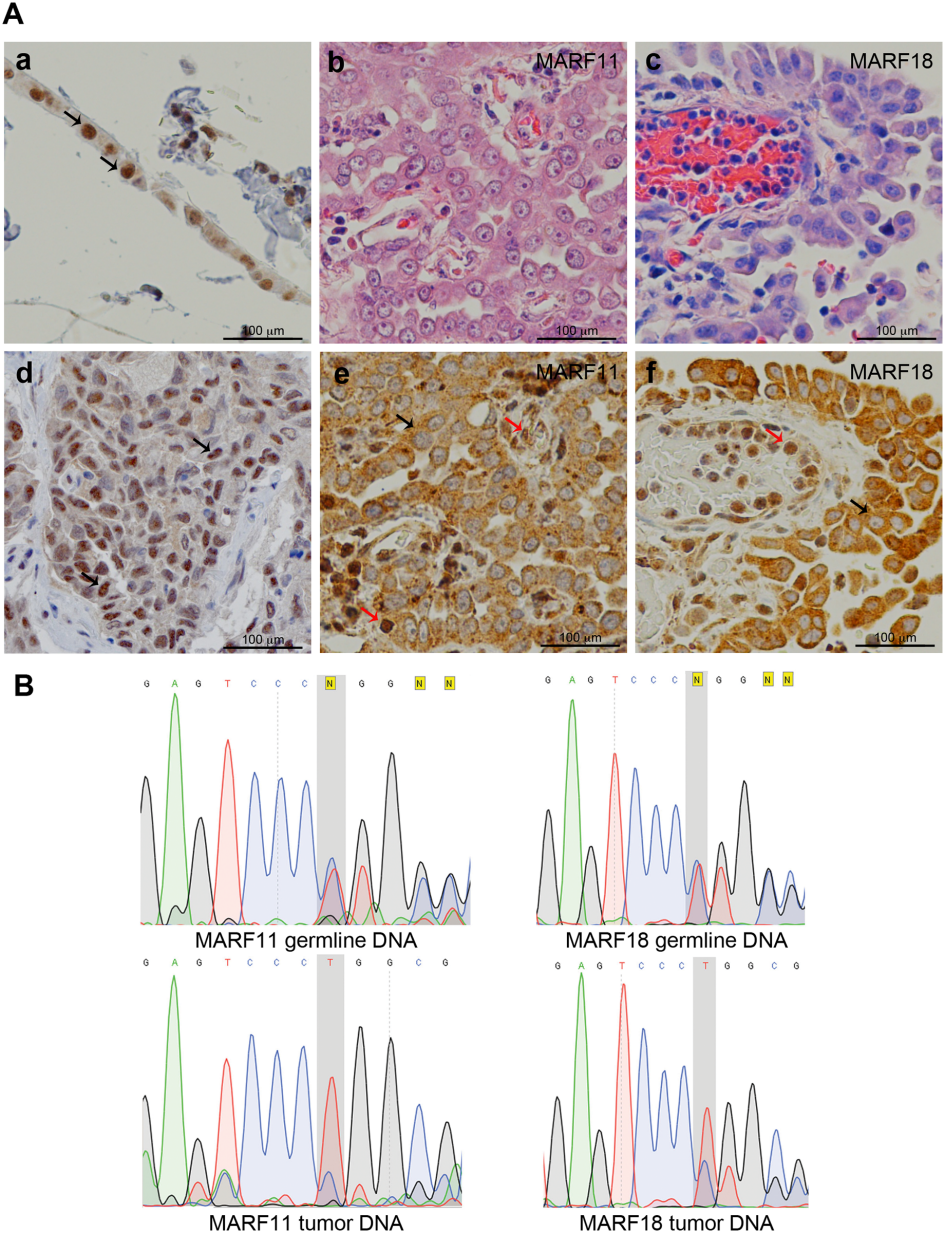
BAP1 cytoplasmic staining and LOH in MM biopsies. **(A)** Representative histology (Hematoxylin and Eosin staining) and BAP1 IHC. Controls: Panel a, normal strip of pleural mesothelial cells; d, MM biopsy containing wild-type BAP1. Note BAP1 nuclear staining and faint cytoplasmic staining; black arrows identify representative normal mesothelial cells in a and MM cells in d. Panels b, c, e and f, BAP1-mutant MM biopsies from MARF11-III-1 (b,e) and MARF18-III-1 (c,f). Note cytoplasmic BAP1 staining and absence of nuclear staining in MM cells; black arrows identify representative tumor cells indicating LOH for BAP1. Note that nearby infiltrating “normal” lymphocytes and endothelial cells show nuclear BAP1 staining (red arrows) as they retain one normal BAP1 allele. Original magnification, 400X. **(B)** *BAP1* sequencing of germline and tumor DNAs from MARF11-III-1 and MARF18-III-1 reveals heterozygosity in germline DNA and LOH in tumor cell DNA. Top panels, germline DNA from both patients shows a ‘C’ deletion (grey shadowed area): the wild-type allele and the mutant allele show the same peak intensity indicating a heterozygous mutation. Bottom Panels, tumor cell DNA, from both patients, shows a homozygous C deletion. The electropherogram of tumor cell DNA shows that the allele with the C deletion has a higher peak, indicating that only the mutant allele is present in the tumor cells. The lower peak is likely generated by the wild type allele of some contaminating normal cells. DNA sequence of wild-type—AGTCCCCTGGC; DNA sequence of mutant—AGTCCCTGGCG.

The heterozygous *BAP1* mutation found in the four probands (MARF2-IV-2, MARF11-III-1, MARF18-III-1, MARF40-III-1) causes a frame shift deletion (*c*.*1717_1717delC*) where Leu→Trp, leading to a premature stop codon, which occurs two amino acids downstream. The resulting truncated BAP1 protein is 573 amino acids long and lacks the nuclear localization signal. Therefore, this mutation is expected to result in the cytoplasmic localization of the truncated BAP1 protein. Immunohistochemistry (IHC) of MM tissues showed only cytoplasmic BAP1 staining and lack of nuclear BAP1 staining, suggesting that the remaining wild-type allele had also become altered in the tumor cells (Fig 2A). Loss of heterozygosity (LOH), a common somatic rearrangement of the BAP1 gene commonly found in MM and other tumors [26], was confirmed by tumor tissue DNA sequencing in MARF11-III-1 and MARF18-III-1, for which tumor tissue was available (Fig 2B). Although MM tumor tissues were not available for MARF2-IV-2 and MARF40-III-1, IHC of MARF2-IV-2 giant bone tumor tissue indicated lack of BAP1 nuclear staining (S2 Fig), supporting presence of LOH, as our previous studies have shown a 100% correlation between LOH and lack of nuclear staining [26]. These data confirmed that the malignancies observed in the four probands are associated with BAP1 alterations.

### An extended kindred of BAP1 cancer syndrome is associated with the *BAP1* c.1717_1717delC mutation

The extent of the shared haplotypes surrounding the *BAP1* gene, between the four MARF probands carrying the *c*.*1717_1717delC BAP1* mutation, indicated that these, presumably unrelated individuals, had a common ancestor. Therefore, we performed extensive genealogical surveys of their families. Genealogical searches using historical census data, birth and death certificates, hospital records, and information from the Ancestry.com database generated data to construct a large pedigree, which we named “K4” kindred, with about 80,000 predicted descendants. This pedigree connected the lineage of the four probands carrying the *c*.*1717_1717delC BAP1* mutation and the rare allele rs71651686 to a couple born in Germany in 1710 (male)—whose ancestors were traced back to 1588 in Switzerland and immigrated to Germany in the 17^th^ century—and in 1712 (female). The couple immigrated to North America, where they had at least ten children. One son born in 1748 in Virginia, migrated to Kentucky and was the forebear of probands MARF11-III-1, MARF18-III-1, and MARF40-III-1, while proband MARF2-IV-2 descended from another son, born in Virginia in 1750, who migrated to Ohio (Maps in S3 Fig, show family migration patterns, exact dates of migration are not shown to respect patient’s confidentiality).

**Fig 3:**
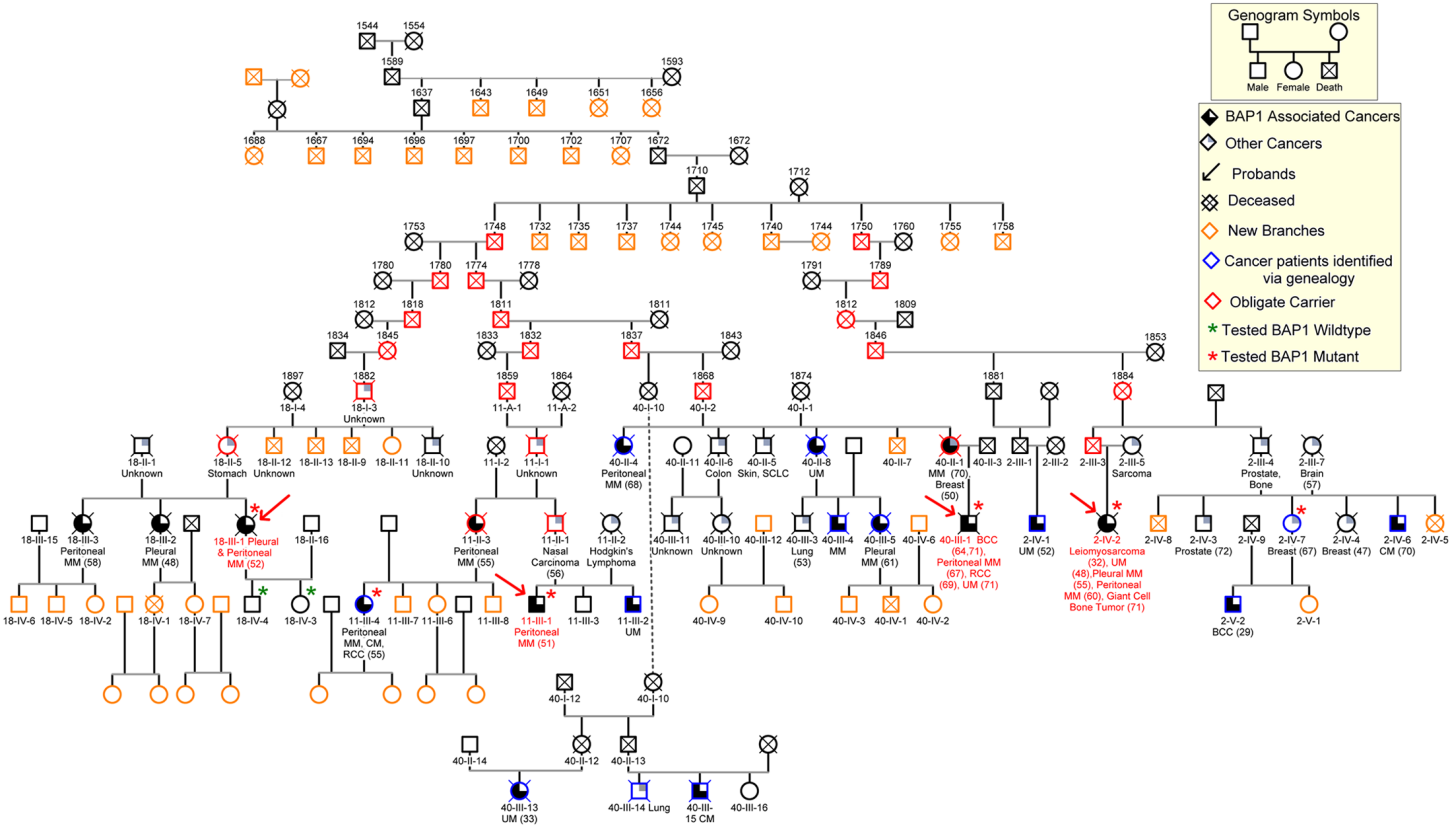
Core of the 106-member nine-generation pedigree, K4. Numbers above symbols represent year of birth; dates of death are not shown to maintain confidentiality. The founding couple was born in Germany in 1710 and 1712, respectively. We were able to trace the origin of the founder male to a Swiss couple, born in 1588 and in 1591, respectively. De-identified patient IDs are shown below the symbols (e.g.11-III-4). Slashed symbols represent deceased individuals. Obligate carriers are indicated with red symbols. All individuals who were tested for presence of germline *BAP1* mutations are indicated with a star: green stars indicate *BAP1* wild type status; red stars indicate *BAP1* mutant carriers. When available, types of malignancies are listed below symbols and ages of diagnoses are indicated in parentheses. Red arrows indicate the probands; blue symbols indicate individuals that we identified through our genealogy search and found to be affected with MM and/or other BAP1 associated malignancies; orange symbols indicate newly identified family branches, that we are actively recruiting into our study, with currently unknown medical history or *BAP1* status. MM, mesothelioma; UM, uveal melanoma; BCC, basal cell carcinoma; RCC, renal cell carcinoma; CM, cutaneous melanoma; SCC, squamous cell carcinoma; all other cancer types are indicated by their full name or anatomical location; “unknown”, the cause of death was cancer, but the histological type was not identified. “other cancers”, malignancies possibly associated with *BAP1* mutations.

The condensed pedigree of 106 individuals with the most relevant information is shown in Fig 3. This pedigree confirmed the relationship among the four *c*.*1717_1717delC BAP1* mutant probands, as indicated by the molecular studies. Most importantly, creating a large family pedigree allowed us to identify new branches of the K4 family (Fig 3, orange symbols) and, among them, individuals affected by cancers characteristic of this syndrome (Fig 3, blue symbols).

## Discussion

Using our screening criteria, we found germline *BAP1* mutations in 18% (4/22) MM patients. The much higher rate of germline *BAP1* mutations that we found in our selected cohort, compared to the percentage (1–2%) found in previous studies among “unselected” MM patients [3, 27], indicates that the selection criteria we used, based on patient’s and family history, are efficient to identify patients with the BAP1 cancer syndrome.

We have identified a heterozygous germline *BAP1 c*.*1717_1717delC* mutation that is responsible for a high incidence of MM, UM, and other cancers among four families (Fig 3 and S2 Table). The absence of a history of asbestos exposure in all four probands suggests that the high penetrance of MM in the *BAP1* mutant families may not require exposure to asbestos (e.g., at least professional exposure or identifiable environmental exposure, for a critical analysis of human carcinogen see ref. [28]). At the same time, only some BAP1 mutant families experience a high prevalence of MM, suggesting that in some families a low level of asbestos exposure may be a co-factor [28], while other families have higher prevalence of different tumor types, such as melanomas, etc. Indeed, we recently published that BAP1+/- mice were susceptible to develop MM when exposed to very low levels of asbestos, levels that rarely trigger MM in wild-type mice [29]. Thus, it is possible that exposure to low levels of asbestos may have triggered MM also in some of these individuals carrying germline *BAP1* mutations. This hypothesis is difficult to verify in non-professionally exposed individuals, due to the intrinsic difficulty of assessing low asbestos exposure levels.

Through combined molecular and genealogical approaches we determined that these four probands, carrying the *BAP1 c*.*1717_1717delC* mutation, are related to a common ancestor, traced through nine generations. The resulting members of the kindred “K4” are genetically high-risk individuals for developing MM and other BAP1-associated malignancies. K4 is the largest pedigree of its kind for the BAP1 cancer syndrome and can be used in genetic counseling for predictive testing (Fig 3). This family pedigree is still in progress as new information is added to the pedigree as it is acquired. As more branches of the family will be identified they will be offered testing for *BAP1*.

We performed a comprehensive analysis of all reported *BAP1* germline mutations, which we compiled in S4 Fig (see also S3 Table for reference list) to see if additional individuals had been reported in the literature to carry this particular mutation. We found that the *c*.*1717_1717delC BAP1* mutation identified in our K4 was recently reported by Cebulla et al. in an apparently unrelated family from Ohio [30]. Although it is possible that the same mutations arose independently in multiple individuals, our genealogic data tracing the migration of K4 throughout the US indicated that they migrated through Ohio (S3 Fig); thus it is likely that the individuals described by Cebulla et al. [30] are related to the K4 family reported here. This hypothesis will be investigated and, if proved correct, the family will be entered into the K4 pedigree (Fig 3). Likewise, recurrent mutations in other parts of the *BAP1* gene –i.e., not the *c*.*1717_1717delC* reported in this manuscript– have been found in several different families, see S4 Fig and S3 Table, suggesting the possibility that also those families are related.

*BAP1* mutations usually cause cancer after the peak of the reproductive age is passed [18]. Since these mutations do not appear to have deleterious effects, other than causing cancer in individuals after the reproductive age [18], they are not negatively selected for, and instead they are transmitted across generations, as we discovered and reported here.

Here we demonstrate and propose that a combination of a carefully taken patient and family history, together with modern molecular genetics and genealogical studies can be used to identify potential carriers of germline *BAP1* mutations and to build large family trees. These family trees can be used to identify additional branches of the family that separated over the course of time, and that may be still carrying germline *BAP1* mutations, and that will benefit from this information. Indeed, as shown in Fig 3, using this approach we identified several new branches of the K4 family affected by MM and by other BAP1 cancer syndrome-associated malignancies (those highlighted in blue in Fig 3). Specifically, through genealogical analyses we identified the ancestors of these four probands, then, by “reverse genealogy”, we identified additional descendants of the original ancestors and, among them, patients with multiple BAP1 related malignancies, who have or are undergoing BAP1 testing (Fig 3). For example, from MARF 11-III-1 we identified his father MARF 11-II-1 who had nasal carcinoma and his sister, MARF 11-II-3, who had peritoneal MM and her four children. One of them had melanoma, renal carcinoma, and peritoneal MM –diagnosed almost simultaneously at age 55. She tested positive for BAP1 germline mutation (Fig 3). Her relatives and descendants are now been closely monitored for early cancer detection and are being tested for germline BAP1 mutations. Similarly, the identification of the ancestors of proband MARF40-III-1 and proband MARF2-IV-2 allowed us to identify additional descendants of the original ancestors, including branches of these families with multiple BAP1 related malignancies who have/are undergoing BAP1 testing (Fig 3). Once new branches of the family carrying germline *BAP1* mutations are identified, these family members, affected by MM, can be informed that their malignancy is usually associated with significantly longer survival than those occurring sporadically [18]. Those that do not have disease and are found to be carriers of *BAP1* germline mutations can be followed for early cancer detection [7]. Those who do not have disease and who did not inherit the mutation can be reassured they and their descendants are not at higher risk of malignancy than the general population.

Early diagnosis and treatment may be partly responsible for the significantly improved prognosis of MM in germline *BAP1*-carriers [18]. Therefore, we have, and are, enrolling several BAP1 family members in a prospective study that includes yearly dermatological and ophthalmological evaluations for early detection of CM and UM, which are curable malignancies when detected at an early stage. Moreover, novel approaches based on biomarkers studies are being investigated in these families to improve early detection for MM and other cancers, as almost all malignancies are more susceptible to therapy when detected at an early stage. As we learn more about the pathways that are altered in individuals carrying germline *BAP1* mutations, novel target approaches will be developed to benefit them.

In summary, it is clinically relevant to identify carriers of *BAP1* mutations and patients who developed cancer in a background of germline *BAP1* mutations. Because *BAP1* germline mutations are passed through multiple generations, building genealogical trees, as the one shown in Fig 3, will lead to the identification of many more families who carry these mutations and who will benefit from this information.

## Methods

### Study oversight

Written informed consent was received from all patients. Collection and use of patient information and samples were approved by the IRB of the University of Hawaii (IRB no.14406).

### Patient recruitment

Patients were recruited based on family histories suggestive of the BAP1 cancer syndrome. Inclusion criteria were: 1) age at MM (either pleural or peritoneal MM) diagnosis less than 70 years; 2) presence of at least one other MM in first-degree relatives across two generations, and/or presence of at least one other of the following BAP1 cancer syndrome-associated malignancy (UM, CM, RCC, BCC or cholangiocarcinoma) in either the proband or a first-degree relative or history of multiple cancers in the first-degree relatives. Twenty-nine MM patients were identified –all epithelioid MMs– and 22 of them agreed to participate in the study and submitted blood for DNA isolation and *BAP1* sequencing. When available, tumor tissues from these individuals were collected for detection of somatic BAP1 status, according to IRB guidelines.

### Salient clinical phenotypes of the four probands and MM families studied

MARF2-IV-2 proband was diagnosed with uterine leiomyosarcoma at age 32, UM at age 48, pleural MM at age 55, peritoneal MM at age 60, giant cell bone tumor at age 71 and died at 72.

MARF11-III-1 proband was diagnosed with peritoneal MM at age 51 and is presently 57. His brother (MARF11-III-2) was diagnosed with UM at age 51 and is presently 61.

MARF18-III-1 proband and her two older sisters were diagnosed with MM before age of 58. The proband had both pleural and peritoneal MM and survived seven years from diagnosis. One of her sisters (MARF18-III-2) had pleural MM and died from complications of treatment; the other sister (MARF18-III-3) had peritoneal MM and survived nine years from diagnosis.

MARF40-III-1 proband was diagnosed with four of the malignancies that have been conclusively demonstrated to be part of the BAP1 cancer syndrome: two basal cell carcinomas (BCC) diagnosed at ages 64 and 71, peritoneal MM at age 67, RCC at age 70, and UM at age 71. He died of pneumonia at age 72. The proband’s mother (MARF40-II-1) was diagnosed with breast cancer at age 50 and peritoneal MM at age 70; one maternal aunt (MARF40-II-4) died at age 68 of peritoneal MM, another maternal aunt was diagnosed with UM (MARF40-II-8); a cousin (MARF40-III-5) was diagnosed at age 71 with pleural MM.

Additional information can be found in S2 Table.

### *BAP1* sequencing

Genomic DNA was extracted from whole blood or from tumor tissues and the *BAP1* gene was directly amplified by PCR in its entirety as previously described [3]. Briefly, DNA was extracted using either DNeasy Blood & Tissue Kit (QIAGEN, Hilden, Germany), or QiAamp DNA Micro Kit (Qiagen) following the manufacturer’s instructions. Advantage2 DNA polymerase (Clontech) was used with each pair of primers under the following conditions: denaturation at 95°C for 2 min; then five cycles of 95°C for 1 min and 68°C for 1 min; then 35 cycles of 95°C for 30 s, 63°C for 30 s and 68°C for 30 s; concluding with 68°C for 5 min. PCR products were gel-purified and Sanger sequenced. Genomic *BAP1* PCR product sizes ranged from 560–670 bp with 100–150 bp overlap between primer sets.

Sequencing was conducted using the ABI 3730XL DNA Sequencer, at the Advanced Studies in Genomics, Proteomics and Bioinformatics facility at the University of Hawaii at Manoa. The *BAP1* mutation *c*.*1717_1717delC* is detected with the following forward primer: CCTCACCCACCCCCAGCA, and reverse primer TGGGAAGAGAGGTCACAA GAAAA. The complete list of primers used for *BAP1* sequencing can be found in reference no.[3].

### SNP genotyping

The four MARF samples (MARF2-IV-2, MARF11-III-1, MARF18-III-1, MARF40-III-1), and four control samples from the University of Hawaii Cancer Center were genotyped on the Illumina OmniExpress (OE) array by AROS Applied Biotechnology (Aarhus, Denmark). We imported genotype data into R and converted it into snpStats genotype data format. The orientation and uniqueness of SNP positions was determined by comparing to the human hg19 reference genome (http://hgdownload.cse.ucsc.edu/goldenPath/hg19/chromosomes) using in-house R scripts and blat. OE SNPs were then filtered to only include those that were uniquely mapped, had 100% call-rate across the eight samples, and minor-allele frequency (MAF) greater than zero across the genotyped samples or in 1000G European samples.

### Genetic and statistic analyses

SNPs and their location in the genome were analyzed using: 1) 1000 Genomes Project (1000G Central European or British ancestry: CEU+GBR; n = 205; 2) UK10K Project (n = 3781); 3) NHLBI Exome Sequencing Project (ESP; n = 4300). Reference 1000G Illumina Omni 2.5M genotype data (Omni2.5) for 2141 samples from 19 worldwide sample populations was combined for analysis with the OE data. Quality control metrics such as call-rate, MAF, alternate-allele frequency (AAF), and Hardy-Weinberg Equilibrium (HWE) *P*-value were calculated across SNPs and samples in each population. We performed principal component analysis (PCA) and genome-wide identity-by-descent (IBD) analysis of OE+Omni2.5 using the R statistics package SNPRelate (http://github.com/zhengxwen/SNPRelate), after running LD-based pruning to remove redundant/correlated SNPs[22, 23]. PCA is a method used to reduce a high-dimensional dataset consisting of many correlated variables down to a smaller set of uncorrelated variables termed principal components (PCs) and is commonly used to investigate the ancestral ethnicity and geographic origin of a set of samples using genome-wide genotype data [20]. Phased haplotypes for complete chromosome 3 genotype data were estimated using SHAPEIT2 and IBD shared segment analysis run using BEAGLE4 with default parameters.

### Immunohistochemistry

BAP1 staining was performed as previously described [3]. Briefly, Formalin-fixed paraffin embedded tissue sections were first deparaffinized and rehydrated, then immersed in 3.0% hydrogen peroxide in methanol for 10 min at room temperature (RT) to block endogenous peroxidase activity. Heat antigen retrieval was conducted at 121°C for 5 min in 0.01 M citrate buffer (pH 6.0). Staining was performed using the Vectastain Elite ABC Kit and the C-4 monoclonal mouse anti-BAP1 antibody (Santa Cruz, CA) diluted 1/100.

### Genealogical studies

A professional genealogist (H.H.) investigated the common ancestors of the four MM patients carrying germline *BAP1* deletion *c*.*1717_1717delC*. Data were obtained from Ancestry.com (http://www.ancestry.com), historical census, birth, death certificates, and hospitals.

## Supporting Information

S1 Table Genome-wide IBD analysis. IBD coefficients between the four probands and four unrelated individuals (UHCC samples) are shown. k0 = probability of sharing zero IBD; k1 = probability of sharing one IBD; kinship = estimated kinship coefficient. MARF11-III-1 and MARF40-III-1 show the highest kinship coefficient.
(PDF)

S2 Table Documented malignancies in the K4 kindred. Bold and italicized, probands of the four related families carrying germline *c*.*1717_1717delC BAP1* mutation. Age of diagnosis and survival time are indicated, if available. ^#^, years of survival from cancer diagnosis; *, individuals tested for presence of germline BAP1 mutation that were found carrying the BAP1 mutation; ^ob^, obligate carriers. ^, Cause of death, cancer, histological type not identified.
Abbreviations: dx, diagnosis; LM, leiomyosarcoma; UM, uveal melanoma; MM, mesothelioma; GCTB, giant cell tumor of the bone; CM, cutaneous melanoma; BCC, basal cell carcinoma; SCC, squamous cell carcinoma; RCC, renal cell carcinoma; all other cancer types are indicated by their full name or by anatomical location.
(PDF)

S3 Table Comprehensive compilation of all reported *BAP1* germline mutations. (PDF)

S1 Fig Principal component analysis (PCA) clusters MARF samples with 1000 Genomes Project European ancestry population samples. Plot of top two principal components from PCA performed using genotype data for 94,510 LD-pruned SNPs with the MARF samples (larger yellow diamond symbols) combined with: **(A)** all 1000 Genomes Phase 1 Project samples, **(B)** European ancestry samples. Note, the four MARF probands overlap with CEU and GBR samples. Legend at top shows plotted points’ symbols to denote larger groupings of world-wide sample populations based on similarity of ancestral populations’ geographic origins, with point color used to separate different population samples. Ancestry abbreviations: UH = University of Hawaii (MARF = proband and control samples); AFR = African (ACB = African Caribbean in Barbados; ASW = African Ancestry in Southwest US; LWK = Luhya in Webuye, Kenya; YRI = Yoruba in Ibadan, Nigeria); AMR = Americas (CLM = Colombian in Medellin, Colombia; MXL = Mexican Ancestry in Los Angeles, California; PEL = Peruvian in Lima, Peru; PUR = Puerto Rican in Puerto Rico); ASN = East Asian (CDX = Chinese Dai in Xishuangbanna, China; CHB = Han Chinese in Bejing, China; CHS = Southern Han Chinese, China; JPT = Japanese in Tokyo, Japan; KHV = Kinh in Ho Chi Minh City, Vietnam); EUR = European (CEU = Utah residents with Northern and Western European ancestry; FIN = Finnish in Finland; GBR = British in England and Scotland; IBS = Iberian populations in Spain); SAN = South Asian (GIH = Gujarati Indian in Houston, TX).
(TIF)

S2 Fig BAP1 IHC of MARF2-IV-2 giant bone tumor tissue. Black arrows show representative tumor cells. Original magnification, 400X.
(TIF)

S3 Fig Migration pattern of the K4 kindred from Europe throughout North America. The four probands lineages (MARF2, MARF11, MARF18 and MARF40) descend from a common ancestor born in Switzerland in 1588. The founder couple migrated to Germany in the 1700s and subsequently to US, Pennsylvania. The couple had 10 children that were born in Virginia; two of them, as shown in Fig 3, were the forebears of the 4 probands studied here. The son born in 1748 (Fig 3) was the forebear of MARF11, MARF18 and MARF40 lineages; the son born in 1750 (Fig 3) was the forebear of MARF2 lineage. The figure shows the migration of the original family to North America and the subsequent migration of the four lineages described above across the US States, till the present day. Sequential letters in maps denote the chronological order of lineage migration through different geographical areas during the past 300 years. The specific years of each migration within the US are not shown to maintain confidentiality.
(PDF)

S4 Fig Schematic representation of reported germline *BAP1* mutations associated to increased risk of malignancies, with focus on history of MM. History of MM is found in about 39% of published BAP1 families. Multiple mutations in the same amino-acid and/or identical mutations reported in more than one proband are highlighted. For a complete list of references, see S3 Table. UCH: Ubiquitin Carboxy-terminal Hydrolase domain; H: HCFC1 binding domain; NTD: N-Terminal Domain; NLS: Nuclear Localization Signal. Fs: frame shift; * stop codon; splice: aberrant splicing.
(TIF)
