# Supplementary material for: Combined Genetic and Genealogic Studies Uncover a Large BAP1 Cancer Syndrome Kindred Tracing Back Nine Generations to a Common Ancestor from the 1700s
Source: PLoS Genet. 2015 Dec 18;11(12):e1005633. doi: 10.1371/journal.pgen.1005633 (PMC4686043; doi:10.1371/journal.pgen.1005633)
Supplement: S1 Table — IBD coefficients between the four probands and four unrelated individuals (UHCC samples) are shown. k0 = probability of sharing zero IBD; k1 = probability of sharing one IBD; kinship = estimated kinship coefficient. MARF11-III-1 and MARF40-III-1 show the highest kinship coefficient. (PDF) [file pgen.1005633.s001.pdf]

S1 Table. Genome-wide IBD analysis.

ID1	ID2	k0	k1	kinship
MARF11-III-1	MARF40-III-1	0.9258	0.0742	0.0186
UHCC1	UHCC2	0.9374	0.0626	0.0157
MARF11-III-1	MARF18-III-1	0.9465	0.0535	0.0134
UHCC3	UHCC4	0.9505	0.0495	0.0124
MARF18-III-1	UHCC2	0.9517	0.0483	0.0121
UHCC1	MARF40-III-1	0.9560	0.0440	0.0110
UHCC1	MARF11-III-1	0.9563	0.0437	0.0109
MARF18-III-1	MARF40-III-1	0.9582	0.0418	0.0104
MARF2-IV-2	MARF40-III-1	0.9635	0.0365	0.0091
UHCC3	UHCC1	0.9645	0.0355	0.0089
MARF2-IV-2	UHCC4	0.9671	0.0329	0.0082
MARF11-III-1	UHCC2	0.9674	0.0326	0.0082
UHCC1	MARF2-IV-2	0.9675	0.0325	0.0081
MARF18-III-1	MARF2-IV-2	0.9697	0.0303	0.0076
UHCC3	MARF11-III-1	0.9733	0.0267	0.0067
UHCC1	UHCC4	0.9755	0.0245	0.0061
MARF11-III-1	MARF2-IV-2	0.9768	0.0232	0.0058
MARF40-III-1	UHCC2	0.9812	0.0183	0.0048
MARF11-III-1	UHCC4	0.9816	0.0184	0.0046
UHCC3	MARF18-III-1	0.9841	0.0159	0.0040
MARF2-IV-2	UHCC2	0.9854	0.0138	0.0038
UHCC3	MARF40-III-1	0.9859	0.0141	0.0035
UHCC2	UHCC4	0.9872	0.0128	0.0032

UHCC3	UHCC2	0.9892	0.0108	0.0027
UHCC3	MARF2-IV-2	1.0000	0.0000	0.0000
UHCC1	MARF18-III-1	1.0000	0.0000	0.0000
MARF18-III-1	UHCC4	1.0000	0.0000	0.0000
MARF40-III-1	UHCC4	1.0000	0.0000	0.0000
