# Supplementary material for: Combined Genetic and Genealogic Studies Uncover a Large BAP1 Cancer Syndrome Kindred Tracing Back Nine Generations to a Common Ancestor from the 1700s
Source: PLoS Genet. 2015 Dec 18;11(12):e1005633. doi: 10.1371/journal.pgen.1005633 (PMC4686043; doi:10.1371/journal.pgen.1005633)
Supplement: S2 Table — Bold and italicized, probands of the four related families carrying germline c.1717_1717delC BAP1 mutation. Age of diagnosis and survival time are indicated, if available. #, years of survival from cancer diagnosis; *, individuals tested for presence of germline BAP1 mutation that were found carrying the BAP1 mutation; ob, obligate carriers. ^, Cause of death, cancer, histological type not identified. Abbreviations: dx, diagnosis; LM, leiomyosarcoma; UM, uveal melanoma; MM, mesothelioma; GCTB, giant cell tumor of the bone; CM, cutaneous melanoma; BCC, basal cell carcinoma; SCC, squamous cell carcinoma; RCC, renal cell carcinoma; all other cancer types are indicated by their full name or by anatomical location. (PDF) [file pgen.1005633.s002.pdf]

S2 Table. Documented malignancies in the K4 kindred.

Family	ID	Gender	Survival[#]	Cancer (age at dx)
MARF2	III-4	M		Prostate, Bone
MARF2	IV-1	M	9 (alive)	UM (52)
MARF2	***IV-2****	***F***	***40***	***LM (32), UM (48), pleural MM (55), peritoneal MM (60), GCTB (71)***
MARF2	IV-3	M		Prostate (72)
MARF2	IV-4	F	3	Breast (47)
MARF2	IV-6	M		CM (70)
MARF2	IV-7	F	3 (alive)	Breast (67)
MARF2	V-2	M	3 (alive)	BCC (29)
MARF11	I-1[ob]	M		Cancer^
MARF11	II-1[ob]	M	3	Nasal carcinoma (56)
MARF11	II-3[ob]	F	9	Peritoneal MM (55)
MARF11	***III-1****	***M***	***6 (alive)***	***Peritoneal MM (51)***
MARF11	III-2	M	10 (alive)	UM (51)
MARF11	III-4*	F	1 (alive)	Melanoma, RCC, peritoneal MM (all at 55)
MARF11	III-8	M		BCC
MARF18	I-3[ob]	M		Cancer^
MARF18	II-5[ob]	F		Stomach
MARF18	II-10	M		Cancer^
MARF18	II-12	M		Cancer^
MARF18	***III-1****	***F***	***7***	***Pleural MM/Peritoneal MM (52)***

MARF18	III-2	F	<1	Pleural MM (48)
MARF18	III-3	F	9	Peritoneal MM (58)
MARF40	II-1 [ob]	F	25	Breast (50), peritoneal MM (70)
MARF40	II-4	F	7	Peritoneal MM (61)
MARF40	II-5	M		SCC, Lung
MARF40	II-6	M		Colon
MARF40	II-8	F		UM
MARF40	***III-1****	***M***	***8***	***BCC (64, 71), peritoneal MM (67), RCC (70), UM (71)***
MARF40	III-3	M	16	Lung (53)
MARF40	III-4	M		MM
MARF40	III-5	F	8	Pleural MM (71)
MARF40	III-10	F		Cancer^
MARF40	III-11	M		Cancer^
