# Supplementary material for: Combined Genetic and Genealogic Studies Uncover a Large BAP1 Cancer Syndrome Kindred Tracing Back Nine Generations to a Common Ancestor from the 1700s
Source: PLoS Genet. 2015 Dec 18;11(12):e1005633. doi: 10.1371/journal.pgen.1005633 (PMC4686043; doi:10.1371/journal.pgen.1005633)
Supplement: S3 Table — (PDF) [file pgen.1005633.s003.pdf]

S3 Table. Comprehensive compilation of all reported *BAP1* germline mutations.

Chromosome	Hugo Symbol	Mutation Type	Predicted Protein Change	Sample ID	MM incidence (0 = no, 1 = yes)	Reference (First author, Journal)	PMID
3	BAP1	Frame_Shift_Ins	E402fs	N/A[#]	0	Harbour, Science	21051595
3	BAP1	Nonsense_Mutation	Q684*	Family L	1	Testa, Nat Gen	21874000
3	BAP1	Splice_Site	P147_splice	Family W	1	Testa, Nat Gen	21874000
3	BAP1	Frame_Shift_Ins	L573fs	SP002	1	Testa, Nat Gen	21874000
3	BAP1	Frame_Shift_Ins	S628fs	SP008	1	Testa, Nat Gen	21874000
3	BAP1	Frame_Shift_Ins	Q436fs	Family 1	0	Wiesner, Nat Gen	21874003
3	BAP1	Splice_Site	M687_splice	Family 2	1	Wiesner, Nat Gen	21874003
3	BAP1	Frame_Shift_Ins	V27Cfs	Family 3	1	Wiesner, JCO	23032617
3	BAP1	Frame_Shift_Ins	K25fs	N/A	0	Höiom, Genes Chromosomes Cancer	23341325
3	BAP1	Frame_Shift_Ins	D236fs	Fam562	0	Njauw, PLoS One	22545102
3	BAP1	Nonsense_Mutation	R385*	Fam729	0	Njauw, PLoS One	22545102
3	BAP1	Nonsense_Mutation	R385*	Fam3382	0	Njauw, PLoS One	22545102
3	BAP1	Frame_Shift_Ins	E611fs	Fam3123	0	Njauw, PLoS One	22545102
3	BAP1	Frame_Shift_Ins	A634fs	Fam2734	0	Njauw, PLoS One	22545102
3	BAP1	Nonsense_Mutation	R60*	Fam714	0	Njauw, PLoS One	22545102
3	BAP1	Nonsense_Mutation	L659*	Fam3101	0	Njauw, PLoS One	22545102
3	BAP1	Nonsense_Mutation	Y241*	N/A	1	Cheung, Cancer Genet	23849051
3	BAP1	Frame_Shift_Ins	T253fs	N/A	1	Ribeiro, Ann Oncol	23585512
3	BAP1	Nonsense_Mutation	Q267*	Fum036	1	Abdel-Raman, J Med Genet	21941004
3	BAP1	Splice_Site	H193_splice	N/A	0	Aoude, PLoS One	23977234
3	BAP1	Splice_Site	E602_splice	N/A	0	Aoude, Pigment Cell Melanoma Res	23171164
3	BAP1	Frame_Shift_Ins	D494fs	N/A	0	Aoude, Pigment Cell Melanoma Res	23171164
3	BAP1	Splice_Site	L570_splice	N/A	1	Wadt, Pigment Cell Melanoma Res	22889334
3	BAP1	Nonsense_Mutation	G684*	Fam064	0	Pilarski, Genes Chromosomes Cancer	24243779
3	BAP1	Frame_Shift_Ins	S628Pfs	Fam104	1	Pilarski, Genes Chromosomes Cancer	24243779
3	BAP1	Nonsense_Mutation	Y394*	Fam103	1	Pilarski, Genes Chromosomes Cancer	24243779
3	BAP1	Missense_Mutation	L100P	N/A	0	Maerker, Br J Ophthalmol	24187051
3	BAP1	Splice_Site	A145_splice	1	0	de la Fouchardière, Clin Genet	25080371
3	BAP1	Splice_Site	G220_splice	2	1	de la Fouchardière, Clin Genet	25080371
3	BAP1	Missense_Mutation	T93A	A	0	Popova, Am J Hum Genet	23684012
3	BAP1	Frame_Shift_Ins	M211Hfs	B	0	Popova, Am J Hum Genet	23684012
3	BAP1	Frame_Shift_Ins	D552Ifs	C	1	Popova, Am J Hum Genet	23684012
3	BAP1	Splice_Site	A145_splice	D	0	Popova, Am J Hum Genet	23684012
3	BAP1	Frame_Shift_Ins	D37Efs	E	1	Popova, Am J Hum Genet	23684012
3	BAP1	Frame_Shift_Ins	H224Pfs	F	1	Popova, Am J Hum Genet	23684012
3	BAP1	Splice_Site	P12_splice	G	1	Popova, Am J Hum Genet	23684012
3	BAP1	Frame_Shift_Ins	V550Sfs	H	1	Popova, Am J Hum Genet	23684012
3	BAP1	Nonsense_Mutation	V616*	I	0	Popova, Am J Hum Genet	23684012
3	BAP1	Frame_Shift_Ins	V27Afs	J	1	Popova, Am J Hum Genet	23684012
3	BAP1	Splice_Site	G220_splice	K	1	Popova, Am J Hum Genet	23684012
3	BAP1	Splice_Site	W196_splice	L	0	Popova, Am J Hum Genet	23684012
3	BAP1	Missense_Mutation	L14H	N/A	0	Farley, Mol Cancer Res	23709298
3	BAP1	Nonsense_Mutation	G198*	N/A	0	Gerami, JAMA Dermatol	26154183
3	BAP1	Missense_Mutation	T173C	N/A	1	Klebe S, Biomark Res	26140217
3	BAP1	Frame_Shift_Ins	L573fs	N/A	0	Cebulla, Ophthalmic Genet	25687217
3	BAP1	Frame_Shift_Ins	T16fs	N/A	1	Betti, Genes Chromosomes Cancer	25231345
3	BAP1	Frame_Shift_Ins	D404fs	1	1	Wadt, Clin Genet	25225168
3	BAP1	Frame_Shift_Ins	Q280fs	2	1	Wadt, Clin Genet	25225168
3	BAP1	Nonsense_Mutation	R60*	3	0	Wadt, Clin Genet	25225168
3	BAP1	Nonsense_Mutation	R60*	4	1	Wadt, Clin Genet	25225168
3	BAP1	Nonsense_Mutation	R60*	5	0	Wadt, Clin Genet	25225168
3	BAP1	Frame_Shift_Ins	V171fs	4	0	Gupta MP, JAMA Ophtalmol	25974357
3	BAP1	Frame_Shift_Ins	I214fs	5	0	Gupta MP, JAMA Ophtalmol	25974357
3	BAP1	Nonsense_Mutation	R385*	14	0	Gupta MP, JAMA Ophtalmol	25974357
3	BAP1	Missense_Mutation	N443Y	16	0	Gupta MP, JAMA Ophtalmol	25974357
3	BAP1	Missense_Mutation	S482L	19	0	Gupta MP, JAMA Ophtalmol	25974357
3	BAP1	Frame_Shift_Ins	S582fs	21	0	Gupta MP, JAMA Ophtalmol	25974357
3	BAP1	Frame_Shift_Ins	A634fs	23	0	Gupta MP, JAMA Ophtalmol	25974357
3	BAP1	Nonsense_Mutation	L659*	24	0	Gupta MP, JAMA Ophtalmol	25974357
3	BAP1	Nonsense_Mutation	Y44*	N/A	1	Alakus H, JTM	25889843
3	BAP1	Missense_Mutation	G121R	006090	0	Aoude, Twin Research	25787093
3	BAP1	Missense_Mutation	R150C	002203	0	Aoude, Twin Research	25787093
3	BAP1	Missense_Mutation	P222T	009341	0	Aoude, Twin Research	25787093
3	BAP1	Missense_Mutation	N446I	011937	0	Aoude, Twin Research	25787093
3	BAP1	Missense_Mutation	P519A	052809	0	Aoude, Twin Research	25787093
3	BAP1	Missense_Mutation	V604M	050611	0	Aoude, Twin Research	25787093
3	BAP1	Missense_Mutation	V604M	051964	0	Aoude, Twin Research	25787093
					39%		

[#]N/A, not available
