# Supplementary figures and images for: Combined Genetic and Genealogic Studies Uncover a Large BAP1 Cancer Syndrome Kindred Tracing Back Nine Generations to a Common Ancestor from the 1700s
Source: PLoS Genet. 2015 Dec 18;11(12):e1005633. doi: 10.1371/journal.pgen.1005633 (PMC4686043; doi:10.1371/journal.pgen.1005633)

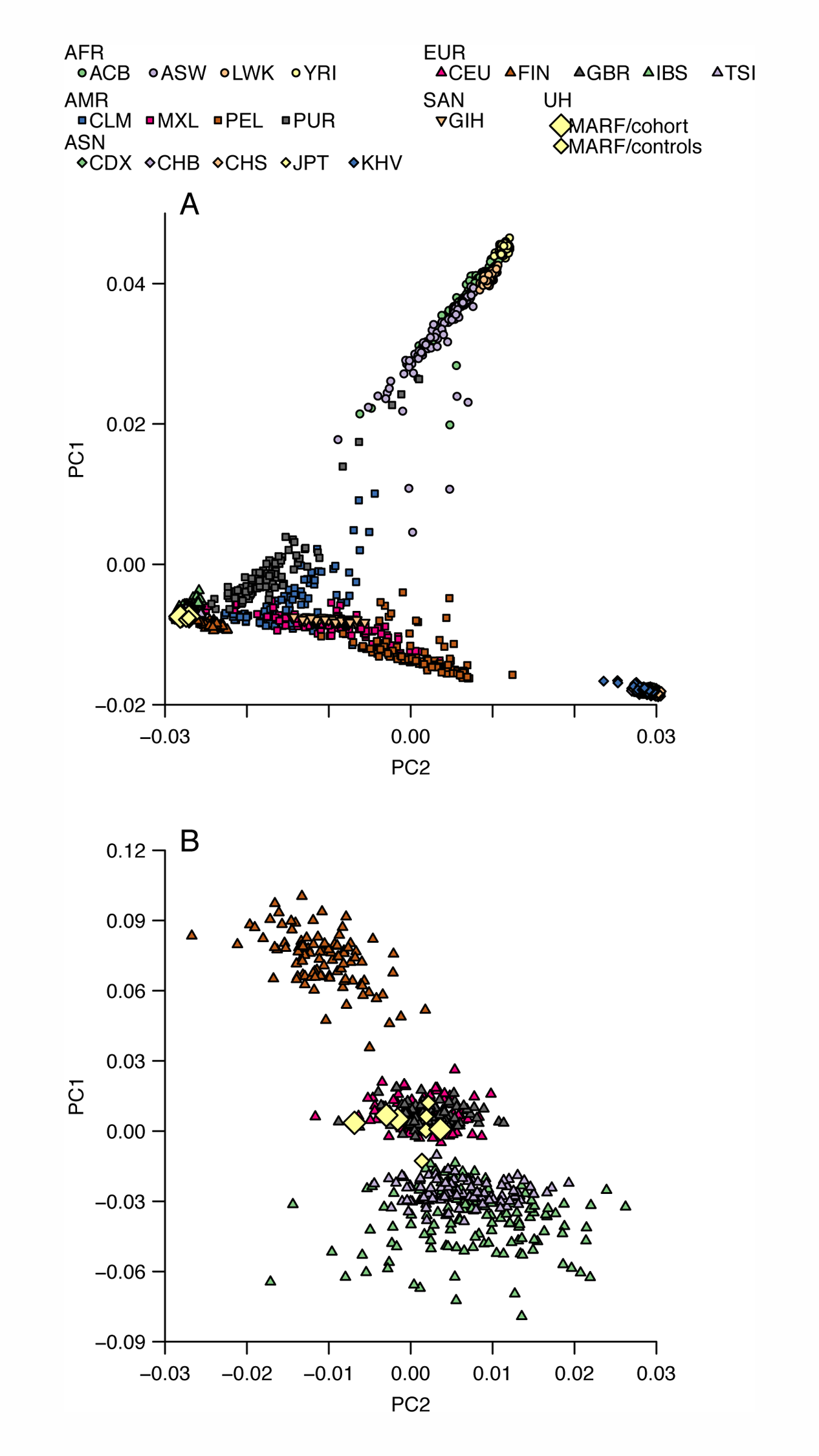

Supplement: S1 Fig — Plot of top two principal components from PCA performed using genotype data for 94,510 LD-pruned SNPs with the MARF samples (larger yellow diamond symbols) combined with: (A) all 1000 Genomes Phase 1 Project samples, (B) European ancestry samples. Note, the four MARF probands overlap with CEU and GBR samples. Legend at top shows plotted points’ symbols to denote larger groupings of world-wide sample populations based on similarity of ancestral populations’ geographic origins, with point color used to separate different population samples. Ancestry abbreviations: UH = University of Hawaii (MARF = proband and control samples); AFR = African (ACB = African Caribbean in Barbados; ASW = African Ancestry in Southwest US; LWK = Luhya in Webuye, Kenya; YRI = Yoruba in Ibadan, Nigeria); AMR = Americas (CLM = Colombian in Medellin, Colombia; MXL = Mexican Ancestry in Los Angeles, California; PEL = Peruvian in Lima, Peru; PUR = Puerto Rican in Puerto Rico); ASN = East Asian (CDX = Chinese Dai in Xishuangbanna, China; CHB = Han Chinese in Bejing, China; CHS = Southern Han Chinese, China; JPT = Japanese in Tokyo, Japan; KHV = Kinh in Ho Chi Minh City, Vietnam); EUR = European (CEU = Utah residents with Northern and Western European ancestry; FIN = Finnish in Finland; GBR = British in England and Scotland; IBS = Iberian populations in Spain); SAN = South Asian (GIH = Gujarati Indian in Houston, TX). (TIF) [file pgen.1005633.s004.tif]

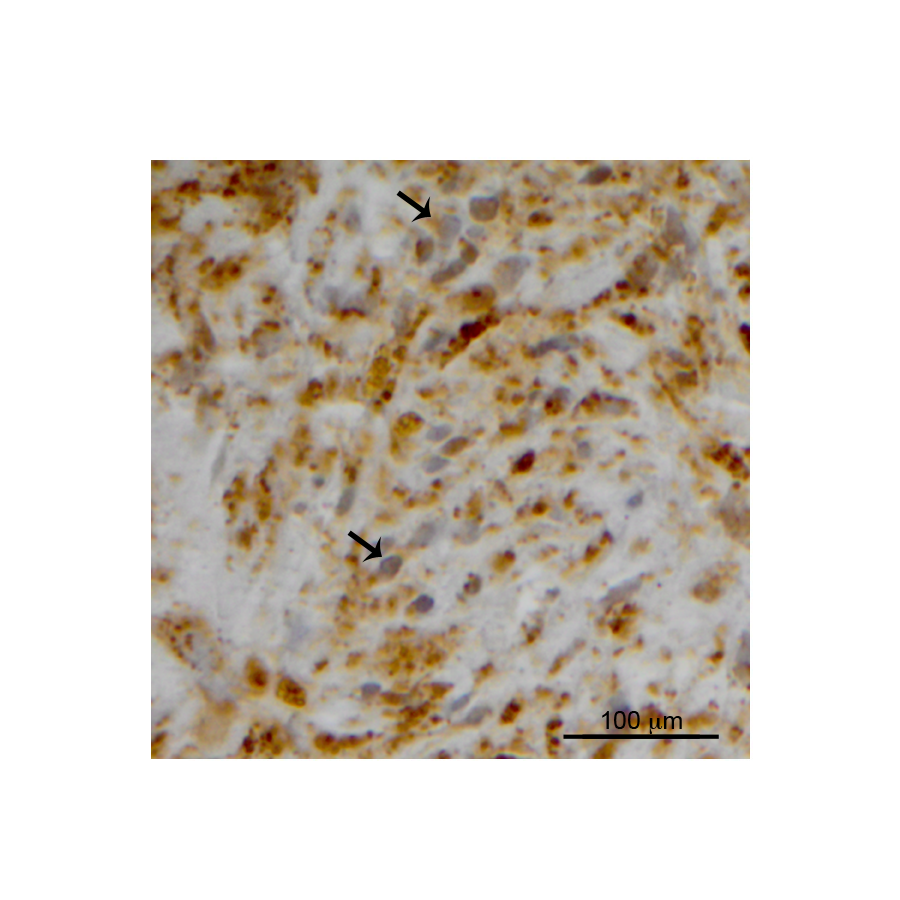

Supplement: S2 Fig — Black arrows show representative tumor cells. Original magnification, 400X. (TIF) [file pgen.1005633.s005.tif]

# MARF2

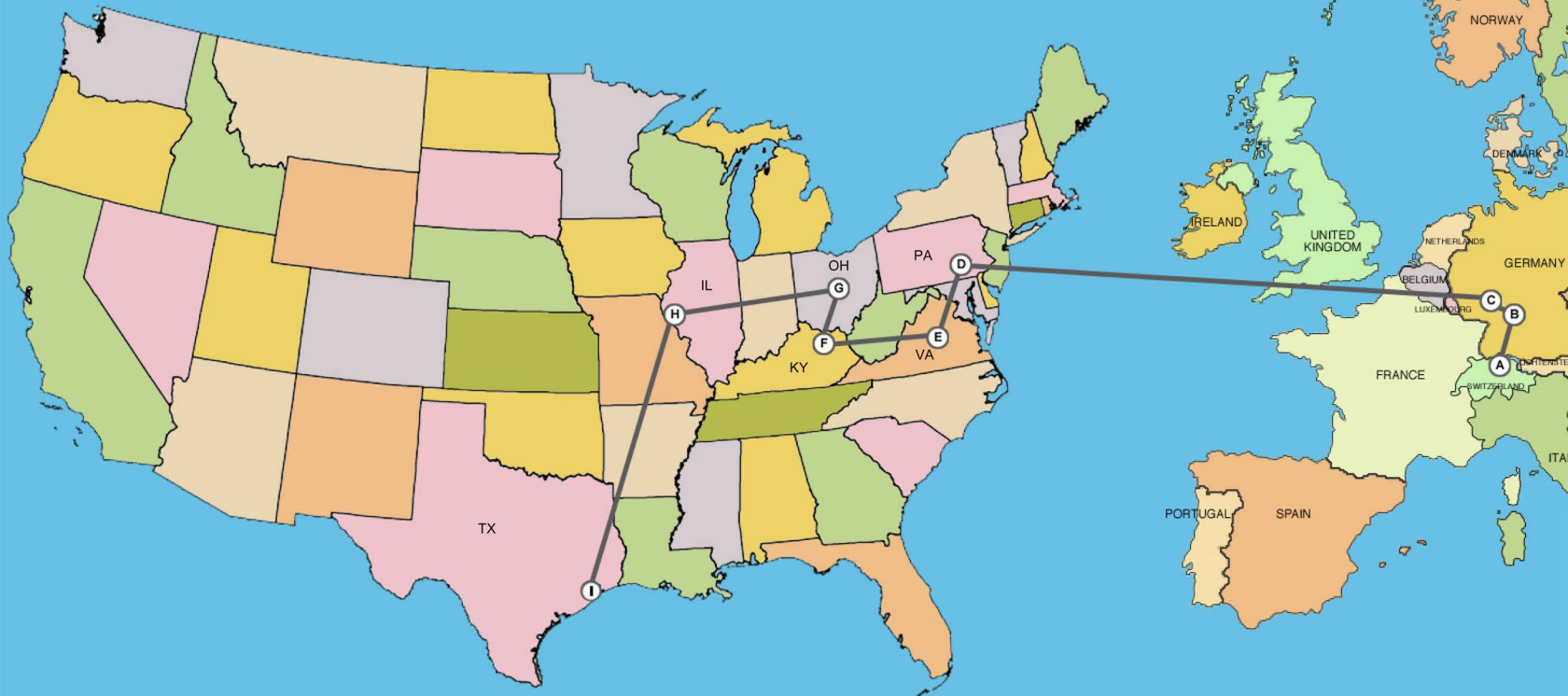

# MARF11

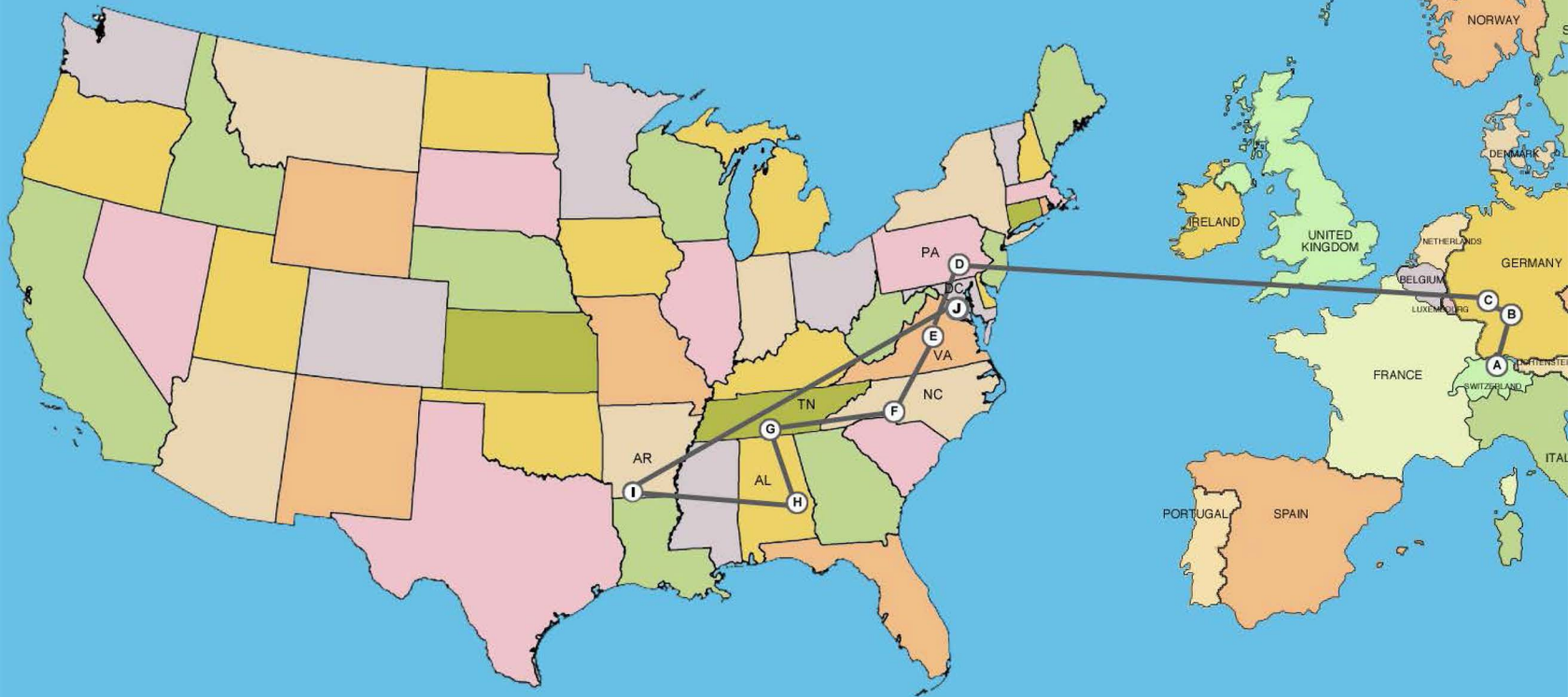

# MARF18

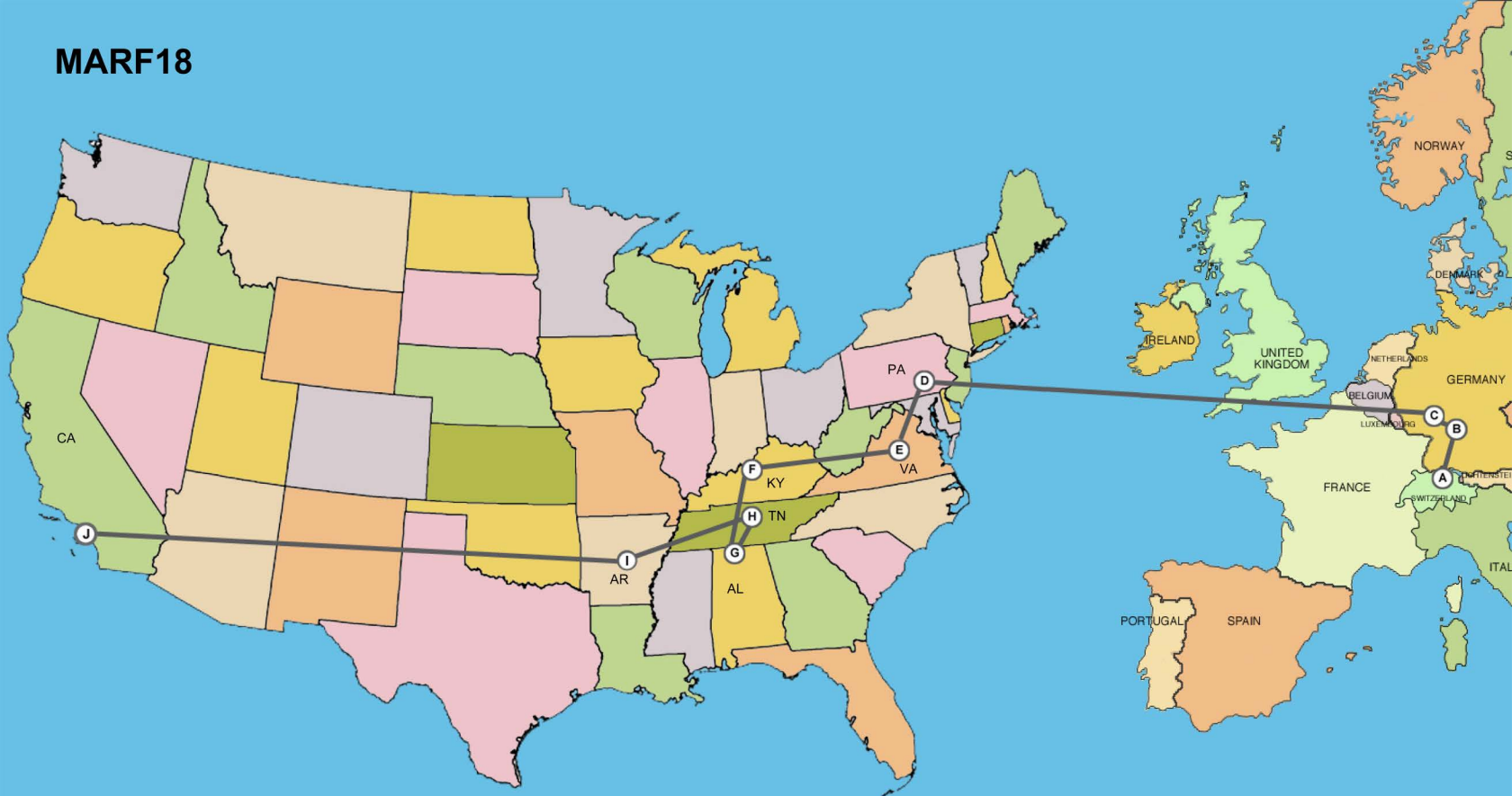

MARF40

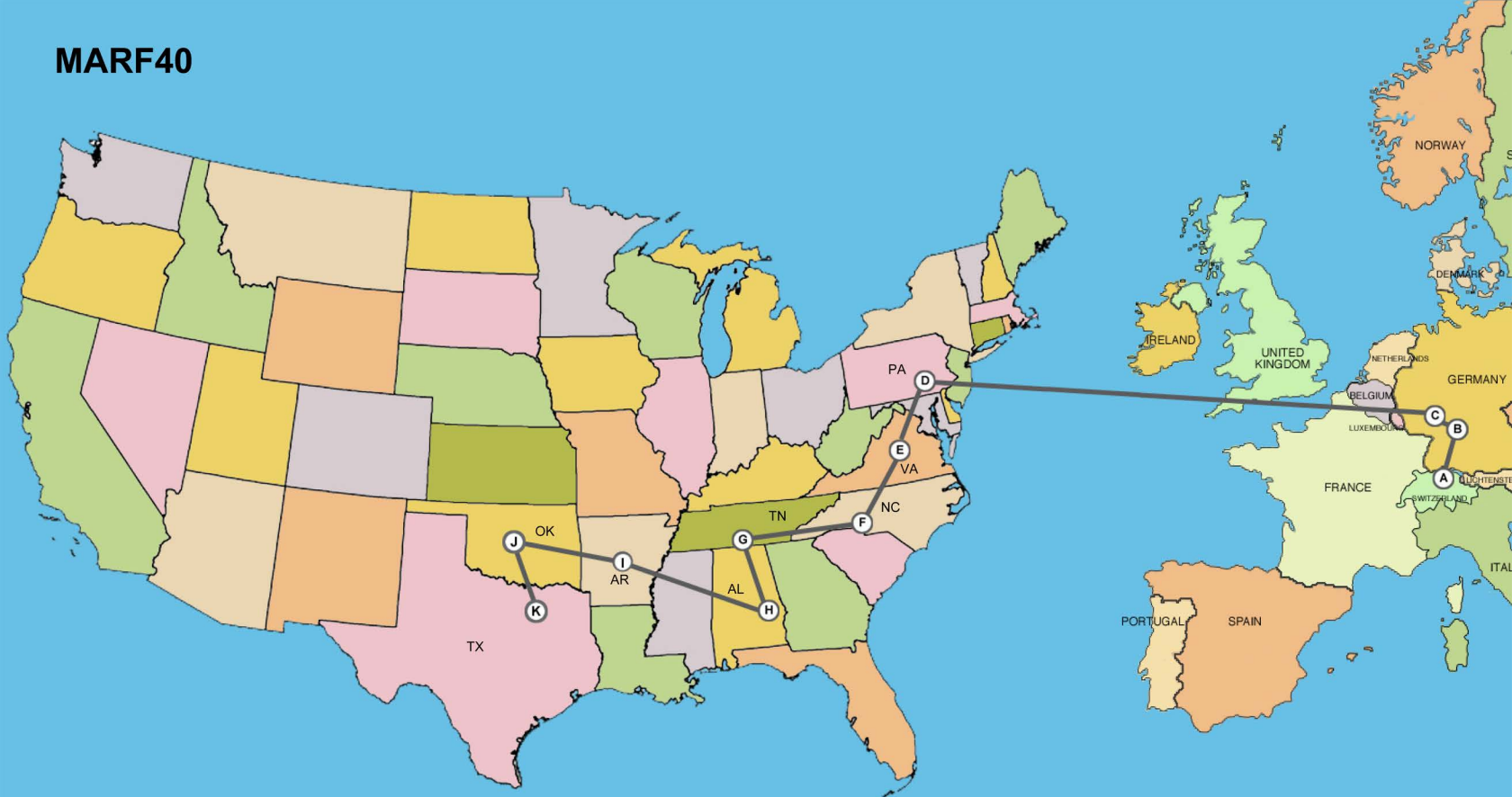

Supplement: S3 Fig — The four probands lineages (MARF2, MARF11, MARF18 and MARF40) descend from a common ancestor born in Switzerland in 1588. The founder couple migrated to Germany in the 1700s and subsequently to US, Pennsylvania. The couple had 10 children that were born in Virginia; two of them, as shown in Fig 3, were the forebears of the 4 probands studied here. The son born in 1748 (Fig 3) was the forebear of MARF11, MARF18 and MARF40 lineages; the son born in 1750 (Fig 3) was the forebear of MARF2 lineage. The figure shows the migration of the original family to North America and the subsequent migration of the four lineages described above across the US States, till the present day. Sequential letters in maps denote the chronological order of lineage migration through different geographical areas during the past 300 years. The specific years of each migration within the US are not shown to maintain confidentiality. (PDF) [file pgen.1005633.s006.pdf]

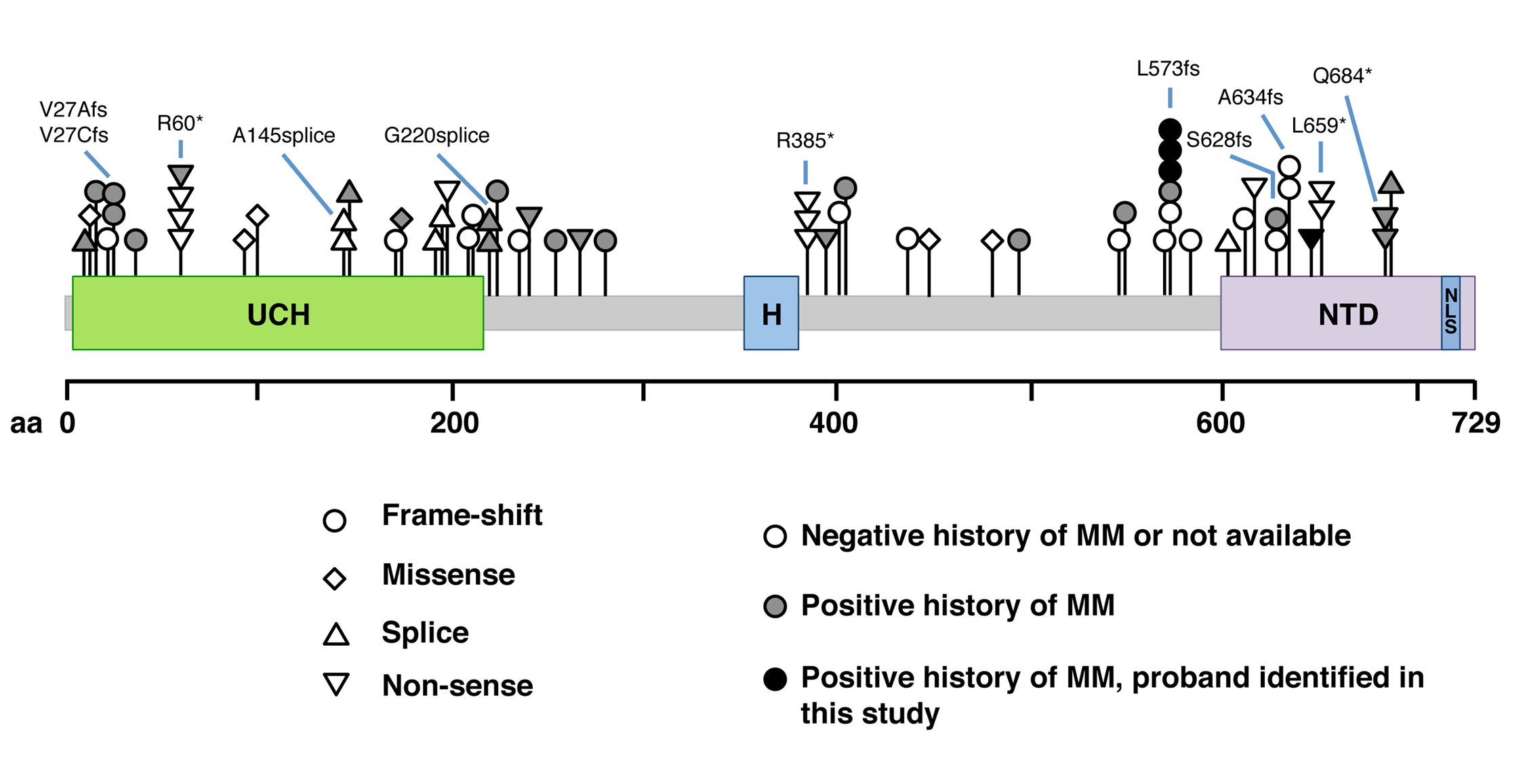

Supplement: S4 Fig — History of MM is found in about 39% of published BAP1 families. Multiple mutations in the same amino-acid and/or identical mutations reported in more than one proband are highlighted. For a complete list of references, see S3 Table. UCH: Ubiquitin Carboxy-terminal Hydrolase domain; H: HCFC1 binding domain; NTD: N-Terminal Domain; NLS: Nuclear Localization Signal. Fs: frame shift; * stop codon; splice: aberrant splicing. (TIF) [file pgen.1005633.s007.tif]
